# Meteorological Factors in Idiopathic Sudden Sensorineural Hearing Loss: A Retrospective Study

**DOI:** 10.7759/cureus.89018

**Published:** 2025-07-29

**Authors:** Athanasios Vlachodimitropoulos, Gerasimos Danielides, Theodoros Stathas, Georgios Batsaouras, Michail Athanasopoulos, Aris I Giotakis, Spyridon Lygeros

**Affiliations:** 1 Otolaryngology - Head and Neck Surgery, University General Hospital of Patras, Patras, GRC; 2 Otolaryngology - Head and Neck Surgery, Hippokration General Hospital, National and Kapodistrian University of Athens, Athens, GRC

**Keywords:** atmospheric pressure, barometric pressure, environmental health, issnhl, meteorology, sudden hearing loss, wind speed

## Abstract

Background and objective: Idiopathic sudden sensorineural hearing loss (ISSNHL), which is an otologic emergency characterized by rapid-onset inner-ear hearing loss without an identifiable cause, remains a clinically urgent but poorly understood entity. While environmental factors have been proposed as potential triggers, prior studies show conflicting results. This study aimed to examine associations between weekly ISSNHL incidence and meteorological variables, including temperature, wind speed, and atmospheric pressure, in a Mediterranean city over a six-year period.

Methods: We retrospectively analyzed 97 ISSNHL cases treated at the University General Hospital of Patras, Greece, from January 2019 to May 2025. Daily weather data were averaged into weekly values and aligned with weekly ISSNHL incidence. Welch’s t-tests and Mann-Whitney U-tests were used to compare weather variables between high-incidence (weeks with >1 case) and low-incidence periods. Lag models were used to assess delayed effects.

Results: No significant seasonal trend in ISSNHL was detected (p=0.973). However, lower wind speeds were consistently associated with higher ISSNHL incidence (p<0.05 across multiple comparisons). A trend toward lower atmospheric pressure in high-incidence windows was also observed. Temperature showed no significant relationships with ISSNHL.

Conclusions: In this Mediterranean setting, ISSNHL incidence was not seasonally patterned but was associated with low wind speed and possibly low atmospheric pressure in the days preceding onset. These findings suggest that calm atmospheric conditions may contribute to ISSNHL risk, potentially via mechanisms involving air quality, vascular reactivity, or inner-ear pressure dynamics.

## Introduction

Idiopathic sudden sensorineural hearing loss (ISSNHL) is an otologic emergency characterized by rapid-onset inner-ear hearing loss without an identifiable cause. Proposed etiologies include viral infection, vascular compromise of the cochlear blood supply, immune-mediated damage, and membrane rupture, yet the precise pathophysiology remains elusive [1]. Environmental and seasonal factors have long been suspected to influence ISSNHL incidence, given analogies to other sudden neurological or vestibular disorders triggered by weather changes [2,3]. However, the evidence regarding seasonal patterns or meteorological triggers in ISSNHL remains inconclusive. Some studies reported higher ISSNHL occurrences during specific seasons or under certain weather conditions; for example, cases correlating with periods of low atmospheric pressure, high temperature, and high humidity (often in spring months) [4]. Others have found no significant seasonal pattern or consistent relationship with weather variables [5,6]. These discrepancies may stem from regional climate differences, study design variability, or the multifactorial nature of ISSNHL.

Against this background, it is important to clarify whether meteorological conditions meaningfully affect ISSNHL incidence. Establishing such links could shed light on underlying pathophysiological mechanisms (e.g., vascular or inflammatory processes modulated by climate change) and potentially guide preventive strategies. The present retrospective study was conducted in Patras, Greece, a Mediterranean city with distinct seasonal weather, to investigate associations between ISSNHL incidence and key meteorological parameters (temperature, atmospheric pressure, and wind speed) over a six-year period. We analyzed both overall seasonal trends and short-term delayed effects of weather variables in the days preceding ISSNHL onset. Our aim was to determine whether fluctuations in these environmental factors correspond with changes in ISSNHL occurrence, thereby providing insight into possible triggers or contributors to this idiopathic condition as well as possible preventive strategies.

## Materials and methods

Study design and setting

This retrospective observational study was conducted at the University General Hospital of Patras, Greece, covering the period from January 1, 2019, to May 30, 2025. The aim was to investigate the relationship between local meteorological conditions and the incidence of ISSNHL. We specifically tested whether weekly variations in weather correlate with increased ISSNHL incidence, whether incorporating a seven-day lag after each week improves predictive associations, and whether seasonal or monthly trends in incidence could be statistically confirmed. Ethical approval was obtained from the Institutional Review Board of the University General Hospital of Patras (#37300), and all data were anonymized prior to analysis.

Patient data collection

ISSNHL cases were identified from hospital admission logs using established clinical criteria as follows: sensorineural hearing loss of sudden onset (≤72 hours), affecting at least three contiguous frequencies with ≥30 dB hearing reduction, and no identifiable cause. Data extracted included the following: date of hospital admission, patient gender, and date of birth. From these, we calculated the weekly incidence of ISSNHL cases and patient age at onset. Demographic analysis was conducted to characterize the population.

Meteorological data collection

Daily meteorological data were sourced from the Meteostat API, using the hospital’s geographic coordinates (latitude 38.2411°, longitude 21.7350°). The following weather variables were retrieved using the Python meteostat library as follows: average daily temperature (tavg {°C}), minimum temperature (tmin {°C}), maximum temperature (tmax {°C}), wind speed (wspd {km/h}), and atmospheric pressure (pres {hPa}). The dataset spanned from January 1, 2019, to May 30, 2025. Daily data were then resampled into weekly averages using Sunday-ending weeks to align with the clinical reporting cycle, as well as to monthly averages to evaluate seasonality. To test for seasonal variation in monthly incidence, data were restricted to the period January 2019 to December 2024, ensuring each calendar month was equally represented over six complete years.

Data processing and lagged case windows

Weekly ISSNHL incidence was calculated and merged with weekly averaged meteorological data. To investigate potential delayed environmental effects, we created lagged incidence windows by extending each weekly observation to include cases occurring in the next seven days. Weeks and time windows were classified as low-incidence weeks/windows (≤1 case of ISSNHL) and high-incidence weeks/windows (>1 case of ISSNHL).

Statistical analysis

Group comparisons (high-incidence vs. low-incidence weeks/windows) were performed for each meteorological variable using Welch’s t-test and Mann-Whitney U-test. Normality was tested using the Shapiro-Wilk test. Categorical seasonality (e.g., monthly incidence) was evaluated using a chi-square goodness-of-fit test. All statistical tests were two-tailed, with significance set at p<0.05. Data preprocessing and statistical analyses were conducted using Python 3.11, with libraries including pandas, numpy, scipy, matplotlib, and seaborn.

## Results

Demographic and case overview

A total of 96 cases of ISSNHL were recorded between January 1, 2019, and May 11, 2025. The mean age of patients at presentation was 49.02±16.53 years (median: 48.85, IQR: 22.67), with an age range of 18 to 83 years. The majority of cases occurred in male patients (55 cases, 57.29%), while 41 cases (42.71%) involved female patients. The weekly mean incidence was 0.29 cases per week (median: 0, IQR: 0), and the two-week mean incidence was 0.57 cases per month (median: 0, IQR: 1).

Resampled meteorological data

The meteorological data, after being resampled into weekly averages, including their mean, median, minimum, and maximum values, are depicted in Table 1.

**Table 1:** Meteorological variables resampled into weekly averages. Tavg_mean: average daily temperature - weekly mean; tmin_mean: minimum daily temperature - weekly mean; tmax_mean: maximum daily temperature - weekly mean; pres_mean: average daily atmospheric pressure - weekly mean; wspd_mean: average daily wind speed - weekly mean

Variables	Mean	Median	Standard deviation	Minimum	Maximum	Unit
Tavg_mean	18.32	17.25	6.36	6.43	31.57	°C
Tmin_mean	14.54	13.85	5.88	1.17	28.17	°C
Tmax_mean	22.69	21.81	6.98	8.86	37.51	°C
Pres_mean	1015.68	1015.05	4.51	1007.8	1028.96	hPa
Wspd_mean	7.96	7.57	2.10	2.31	18.14	km/h

Seasonal and monthly trends

**Figure 1:**
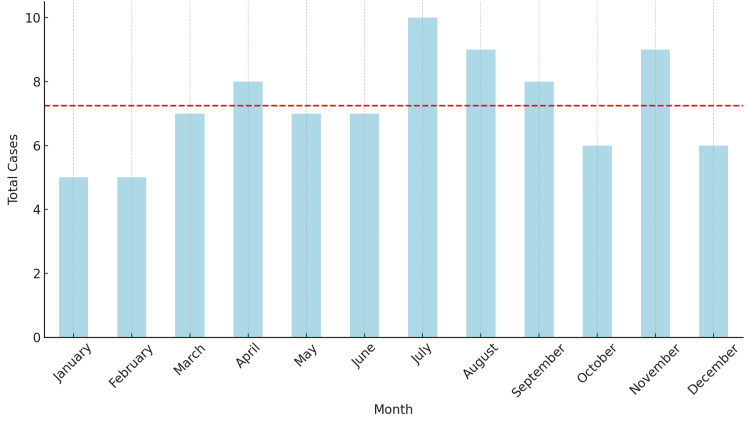
Monthly distribution of ISSNHL cases (January 2019 - December 2024). ISSNHL: idiopathic sudden sensorineural hearing loss

The monthly incidence was analyzed from January 2019 to December 2024, a six-year interval that ensured equal representation across all calendar months (Figure 1). ISSNHL cases ranged from 5 to 10 per calendar month (total: 87, mean: 7.25, median: 7, IQR: 2.25), with July showing the highest counts (10 cases). However, chi-square goodness-of-fit testing showed no statistically significant deviation from a uniform distribution (χ²=3.90, p=0.973).

Weekly meteorological correlation analysis

**Figure 2:**
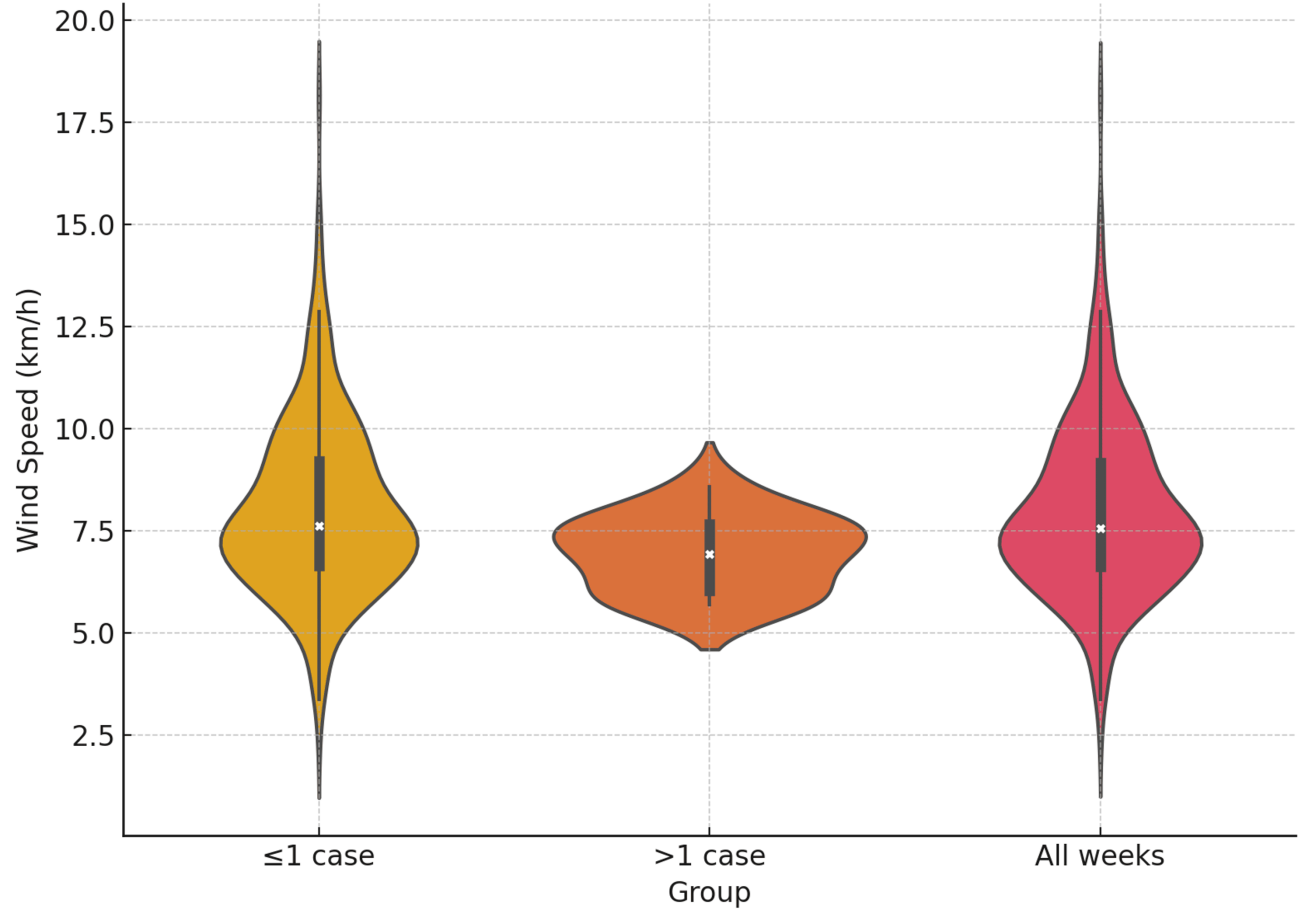
Average weekly mean wind speed of weekly groups.

Weekly ISSNHL incidence was computed and aligned with weekly averaged meteorological variables. Weeks were classified into high-incidence weeks (>1 ISSNHL case {n=14}) and low-incidence weeks (≤1 ISSNHL case {n=314}). Mean wind speed was significantly lower during high incidence weeks when compared to the overall weekly average wind speed (t-test p=0.0013, U-test p=0.0492) (Table 2) and weekly average wind speed in low incidence weeks (t-test p=0.0009, U-test p=0.0401) (Table 3, Figure 2). Mean atmospheric pressure was also significantly lower in both Welch’s t-tests. Temperature variables showed no statistically significant differences (p>0.1 across all tests).

**Table 2:** Comparison of meteorological variables between high-incidence weeks (>1 case) and all weeks. Tavg_mean: average daily temperature - weekly mean; tmin_mean: minimum daily temperature - weekly mean; tmax_mean: maximum daily temperature - weekly mean; pres_mean: average daily atmospheric pressure - weekly mean; wspd_mean: average daily wind speed - weekly mean

Variables	>1 case weeks (mean±SD)	All weeks (mean±SD)	Welch’s t-test p-value	Mann-Whitney U-test p-value
Tavg_mean	19.76±6.20	18.32±6.36	0.410	0.436
Tmin_mean	15.98±6.03	14.54±5.88	0.395	0.411
Tmax_mean	23.99±6.53	22.69±6.98	0.480	0.472
Wspd_mean	6.94±0.92	7.96±2.10	0.0013	0.0492
Pres_mean	1013.64±2.78	1015.68±4.51	0.0192	0.1427

**Table 3:** Comparison of meteorological variables between high-incidence weeks (>1 case) and low-incidence weeks (≤1 case). Tavg_mean: average daily temperature - weekly mean; tmin_mean: minimum daily temperature - weekly mean; tmax_mean: maximum daily temperature - weekly mean; pres_mean: average daily atmospheric pressure - weekly mean; wspd_mean: average daily wind speed - weekly mean

Variable	>1 Case weeks (mean±SD)	≤1 Case weeks (mean±SD)	Welch’s t-test p-value	Mann-Whitney U-test p-value
Tavg_mean	19.76±6.20	18.25±6.37	0.391	0.417
Tmin_mean	15.98±6.03	14.47±5.88	0.375	0.391
Tmax_mean	23.99±6.53	22.64±7.00	0.461	0.453
Wspd_mean	6.94±0.92	8.01±2.12	0.0009	0.0401
Pres_mean	1013.64±2.78	1015.78±4.55	0.0153	0.1261

Delayed-effect analysis

**Figure 3:**
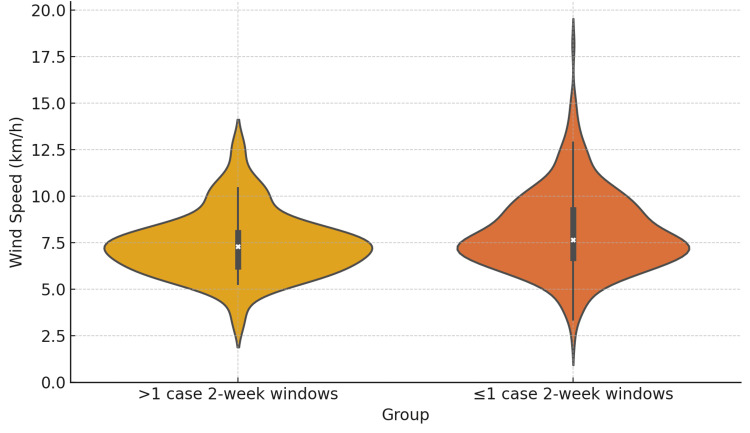
First week's average weekly mean wind speed of high and low incidence two-week windows.

**Figure 4:**
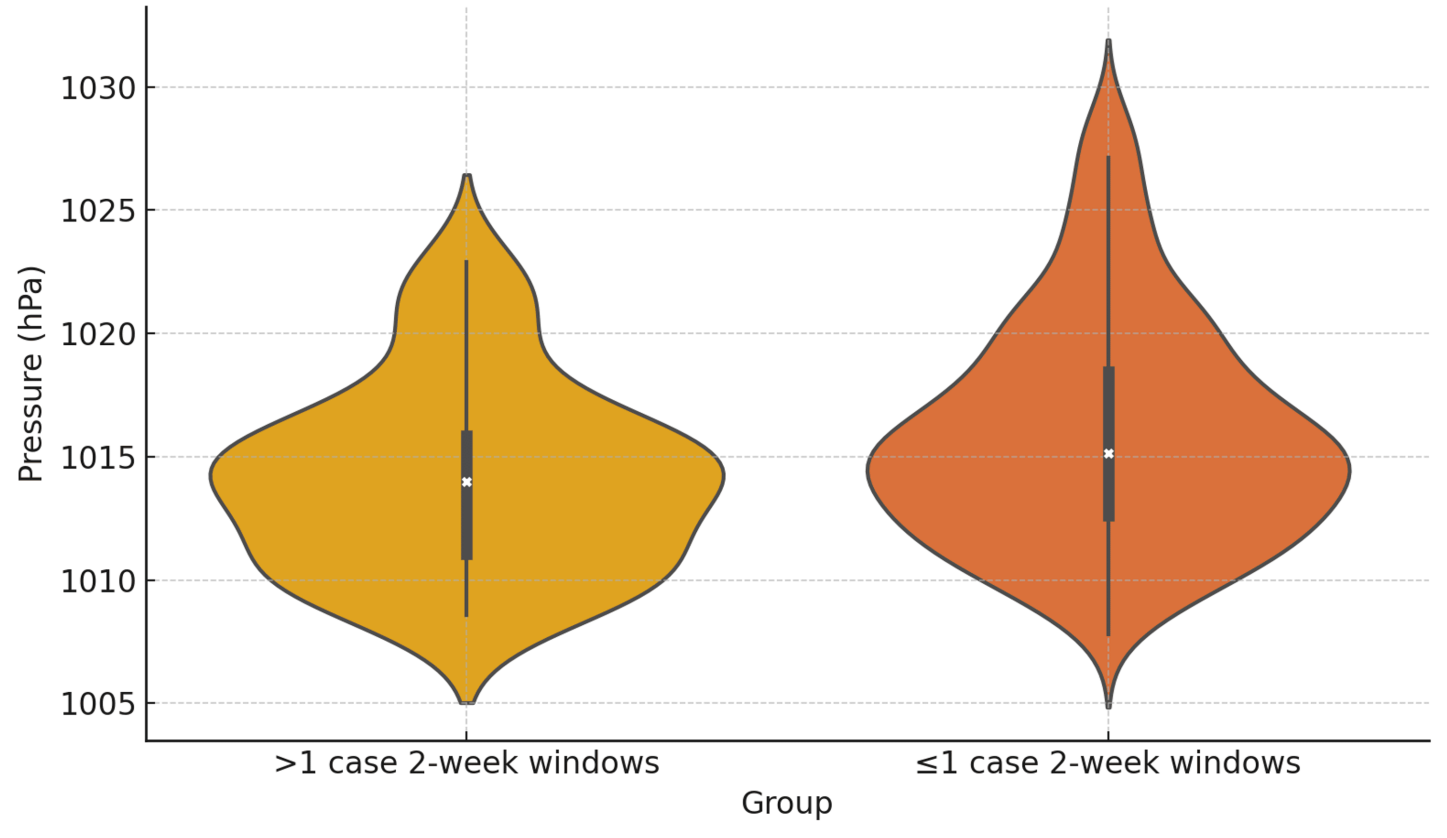
First week's average weekly mean atmospheric pressure of high and low incidence two-week windows.

To investigate whether meteorological factors might precede ISSNHL onset with a short delay, we evaluated seven-day lag windows, each including the week in question plus subsequent days. Based on the median and IQR of the two-week incidence, the 14 windows were classified into high-incidence windows (>1 ISSNHL case {n=44}) and low-incidence windows (≤1 ISSNHL case {n=283}). Wind speed was consistently lower in the first week of high incidence windows compared to low incidence windows (t-test p=0.0226, U-test p=0.0474) (Table 4, Figure 3). A trend toward lower atmospheric pressure was also observed in the seven-day lag model (t-test p=0.0051, U-test p=0.0163) (Table 4, Figure 4). Temperature differences were not statistically significant, though trends for lower temperatures were consistent.

**Table 4:** Comparison of weekly meteorological variables between the first week of high-incidence 14-day windows (>1 case) and low-incidence 14-day windows (≤1 case). Tavg_mean: average daily temperature - weekly mean; tmin_mean: minimum daily temperature - weekly mean; tmax_mean: maximum daily temperature - weekly mean; pres_mean: average daily atmospheric pressure - weekly mean; wspd_mean: average daily wind speed - weekly mean

Variable	First week in high-incidence windows (mean±SD)	First week in low-incidence windows (mean±SD)	Welch’s t-test p-value	Mann-Whitney U-test p-value
Tavg_mean	19.77±6.17	18.08±6.38	0.0982	0.0933
Tmin_mean	15.74±5.40	14.34±5.95	0.1204	0.1190
Tmax_mean	24.32±7.19	22.43±6.93	0.1087	0.1140
Wspd_mean	7.40±1.68	8.06±2.14	0.0226	0.0474
Pres_mean	1014.11±3.77	1015.94±4.57	0.0051	0.0163

Normality testing and justification for statistical methods

The Shapiro-Wilk test demonstrated non-normal distribution in most weather variables, especially among low-incidence weeks and low-incidence windows. Therefore, Mann-Whitney U-tests were reported alongside Welch’s t-tests for robust, assumption-free inference.

## Discussion

In this retrospective six-year analysis, we found no significant seasonal pattern in ISSNHL incidence in Patras. Cases were relatively evenly distributed throughout the year, which is consistent with several prior studies in similar climates. For instance, a large Mediterranean cohort (740 patients) showed no evidence of seasonal clustering of ISSNHL, averaging approximately equal cases per month [6]. Similarly, an earlier study in Northwestern Greece reported no seasonal predilection and no correlation with broad weather “types” [5]. Our findings reinforce that ISSNHL does not uniformly peak in any particular season in temperate regions. This stands in contrast to some reports from other locales; for example, one population study in East Asia noted a higher incidence in autumn [7], while another series observed a predominance in springtime associated with certain climate conditions [4]. Such divergences suggest that any seasonal effect is likely subtle or context-dependent. Overall, the lack of a clear seasonal trend in our data and in multiple international studies implies that large-scale seasonal factors (e.g., temperature extremes or widespread viral outbreaks confined to one season) are not the dominant triggers of ISSNHL in the general population [5,6].

Despite the absence of seasonal variation, we identified specific meteorological associations on shorter time scales. Notably, periods of lower wind speed were consistently linked to a higher weekly incidence of ISSNHL. This inverse association was most pronounced within short lag windows (days immediately preceding hearing loss onset). Interestingly, prior research has yielded mixed observations regarding wind influence. Seo et al. reported that days with ISSNHL tended to have higher mean and maximal wind speeds in the preceding week, with a particularly significant increase in wind velocity about five days before onset [8]. Our results suggest the opposite, ISSNHL cases in Patras were more frequent during calmer periods with reduced wind activity. One potential explanation for this discrepancy is geographic and climatic variation as follows: The coastal Mediterranean climate of Patras differs from the inland climate of the Korean region studied by Seo et al., potentially resulting in different triggering mechanisms (e.g., storm systems versus stagnant high-pressure areas) [8].

Additionally, the analytic approach differed (our study focused on incidence peaks and short-term lags, whereas Seo et al. compared mean conditions on ISSNHL vs. non-ISSNHL days), which might account for the contrasting findings [8]. It is also worth considering that low-wind conditions could be a proxy for other environmental factors - for instance, calm air allows for the accumulation of pollutants or allergens, whereas windy conditions disperse them. Supporting this, a Korean national study found no direct effect of temperature, humidity, or pressure on ISSNHL when controlling for confounders, but did find a strong association with elevated air pollution (notably NO₂ levels) in the two weeks before ISSNHL [9]. Thus, the higher ISSNHL incidence we observed during low-wind periods might reflect increased exposure to airborne pollutants or other agents that accumulate in stagnant air. Unfortunately, our study did not measure air quality, but this connection warrants further investigation.

We also observed a tendency for lower atmospheric pressure to precede increased ISSNHL case numbers, though this relationship was somewhat less robust than the wind speed effect. Low barometric pressure has been proposed as a trigger in prior literature - a multinational review noted that ISSNHL occurrences correlated with periods of low ambient pressure in some settings [4]. Furthermore, Li et al. recently reported from China that deviations in atmospheric pressure (both high and low extremes) were significantly associated with increased hospital admissions for SSNHL [10]. Our findings align with the notion that atmospheric pressure instability might impact the inner ear. Biologically, a falling barometer often signals an incoming weather front and could influence the fluid dynamics or gas content within the inner ear and middle ear. It is conceivable that abrupt pressure changes compromise cochlear microcirculation or oxygenation, akin to the barotrauma and ischemia mechanisms hypothesized in other sudden ear disorders. It is notable that hyperbaric oxygen therapy (involving high-pressure oxygen) is a recognized adjunct treatment for ISSNHL, supporting the idea that oxygen delivery and pressure play a role in inner-ear recovery [11]. By extension, the opposite condition (ambient pressure drops) might transiently reduce oxygen availability or induce microvascular stress in the cochlea, thus precipitating hearing loss in susceptible individuals. This correlation between our epidemiologic data and known treatment rationales provides a plausible pathophysiological link.

Other meteorological variables in our analysis (ambient temperature) did not show a significant independent relationship with ISSNHL incidence. This is in line with several prior studies that, after rigorous adjustment, found no residual effect of temperature or rainfall on ISSNHL rates [12]. In the Taiwanese population study by Lin et al., initial associations with temperature and humidity disappeared after controlling for monthly trends, suggesting those were likely coincidental or seasonally confounded [12]. On the other hand, the comprehensive time-series analysis by Li et al. did detect that extreme cold, wide temperature fluctuations, and high humidity increased ISSNHL risk [10]. We did not observe such effects in Patras’s milder climate, perhaps because truly extreme weather conditions (severe cold swings or very high humidity) are infrequent in this region. Our null findings for these factors do not rule out their importance universally. Still, they indicate that in a Mediterranean setting, temperature and moisture variables were not primary drivers of ISSNHL incidence.

Considering pathophysiological mechanisms, our findings lend themselves to several non-mutually exclusive hypotheses. Weather changes can affect the cardiovascular system, and, by extension, the inner ear’s blood supply [13]. For example, low atmospheric pressure or colder, calmer weather may promote vasoconstriction, changes in blood viscosity, or endothelial dysfunction. ISSNHL patients often harbor vascular risk factors, and evidence of endothelial impairment has been noted in ISSNHL cohorts [14]. Thus, microcirculatory instability in the cochlea - triggered by environmental stress, similar to how temperature or pressure changes can provoke strokes or myocardial infarctions - could lead to the acute ischemia that causes sudden hearing loss [13].

Sudden hearing loss has also been associated with viral insults, including reactivation of herpes simplex virus in some cases [3]. Meteorological conditions might indirectly facilitate viral events - for instance, calm cold air can increase the spread of respiratory viruses, or stress from weather changes might suppress immune surveillance, enabling latent viruses (like herpes simplex virus or varicella-zoster virus) to reactivate in the inner ear. Although this study did not find a winter predominance (which might be expected if new viral infections were a major cause), it remains possible that weather-triggered viral reactivations (not strictly seasonal) contribute sporadically to ISSNHL.

Rapid weather fluctuations can provoke systemic inflammatory responses or allergic reactions in some individuals. Shifts in barometric pressure and temperature have been linked to migraines, joint pain, and other inflammatory flares; similarly, such shifts might induce a pro-inflammatory state in the inner ear or perturb the delicate ion homeostasis of the endolymph [15-17]. Moreover, stable low-wind conditions might elevate exposure to allergens (pollen, mold) and pollutants, triggering inflammation that could damage cochlear hair cells. The association of ISSNHL with air pollution (e.g., NO₂) noted by Choi et al. supports a model in which environmental inflammatory stressors play a role [9]. Our finding that ISSNHL occurred more on days of air stagnation (low wind) further aligns with this concept, hinting that indirect meteorological effects (accumulation of harmful agents) might be a key factor.

Moreover, changes in atmospheric pressure might directly affect the inner ear’s fluids and pressure regulation. The inner ear is somewhat isolated, but via the middle ear and Eustachian tube, barometric changes do influence perilymphatic pressure gradients. A sudden drop in external pressure could lead to a transient relative increase in inner ear pressure or difficulty in pressure equalization, potentially causing stress on the cochlear membranes (not unlike the hypothesized mechanism of perilymphatic fistula in some ISSNHL cases). While speculative, this pressure dysregulation mechanism could operate alongside vascular factors. Interestingly, weather fronts (often accompanied by pressure drops) have been reported to precipitate episodes of Ménière’s disease, indicating that labyrinthine homeostasis is sensitive to such environmental changes [2].

Strengths and limitations

This study adds to the literature by examining ISSNHL in a Mediterranean climate, using six years of data with precise meteorological measurements and considering lag effects of weather variables. However, several limitations must be acknowledged. As an observational study, we can only report associations, not prove causation. Unmeasured confounders, such as air pollution levels, viral infection rates, or individual patient comorbidities, could underlie or modulate the observed weather relationships. In addition, the sample size (limited by the number of ISSNHL cases presenting to our institutions) restricts statistical power for detecting subtle effects of some variables, and may inflate the apparent importance of others due to chance. Furthermore, meteorological data were taken from area-wide stations; individual patients’ true exposures might differ; for example, indoor climate control or travel could modify personal exposure to ambient conditions. We also focused on broad weather parameters; more nuanced factors, such as rapid weather changes (e.g., sudden temperature drops or day-to-day barometric variability), were not separately analyzed and could be relevant triggers. Finally, our findings pertain to one geographic area; results might differ in other regions with different climates (e.g., tropical environments), so caution is needed in generalizing globally.

## Conclusions

In conclusion, our study advances the understanding of ISSNHL by highlighting that acute meteorological factors, rather than broad seasonal trends, may influence its occurrence. The consistent association we observed between calm atmospheric conditions (low wind, and to a lesser extent low pressure) and increased ISSNHL cases suggests that environmental stillness or instability could act as a catalyst in susceptible individuals. These findings concur with some emerging evidence (e.g., impact of barometric pressure) while contradicting others (wind effects), underscoring the complexity of ISSNHL’s triggers. By critically comparing our results with the literature and considering plausible biological mechanisms, we acknowledge that ISSNHL likely arises from a confluence of internal and external factors. Continued interdisciplinary research, spanning otolaryngology, climatology, and epidemiology, is warranted to unravel these factors. Such work will not only deepen our scientific understanding of ISSNHL pathogenesis but also potentially guide preventive strategies and tailored treatments for this enigmatic cause of sudden deafness.
